# Diogenes Syndrome: Identification and Distinction from Hoarding Disorder

**DOI:** 10.1155/2021/2810137

**Published:** 2021-11-25

**Authors:** Carmel Proctor, Sakib Rahman

**Affiliations:** Princess Elizabeth Hospital, The Oberlands Centre, La Rue de La Corbinnerie, St Martin, GY4 6SP Guernsey, UK

## Abstract

“Severe domestic squalor” or Diogenes syndrome is characterised by extreme self-neglect of environment, health, and hygiene, excessive hoarding, squalor, social withdrawal, and a distinct lack of concern or shame regarding one's living condition. This report presents a case of a 51-year-old male admitted to the hospital psychiatric ward following the police removing him from his home. Police officers attended the man's home following the alarm being raised by his stepfather that he had not been seen or heard from in 3 weeks. His home was covered in several feet of rubbish, rotting food, and debris and smelled intensely of rotting mould, urine, and faeces. He was found lying nude on top of garbage with a rug over him. Diogenes syndrome is highly comorbid with psychiatric and somatic disorders, including depression, obsessive-compulsive disorder, personality disorder, and stress. This case report provides a rare opportunity to better understand the distinction of Diogenes syndrome from the closely related condition hoarding disorder. Furthermore, creating an agreed-upon constellation of symptoms representative of Diogenes is essential to creating a formal *Diagnostic and Statistical Manual of Mental Disorders (DSM)* entry, which would facilitate the much-needed development of assessment measures to enable accurate diagnosis and treatment.

## 1. Introduction

The etiopathogenesis of Diogenes syndrome remains unclear, with little consensus regarding the diagnostic criteria [1]. Extant case studies identify and describe the condition as “severe domestic squalor,” with some arguing that this is a better descriptor of the syndrome ([2]; C.R. [3]). Clark, Mankikar, and Gray [4] coined the term Diogenes syndrome, replacing the earlier term senile squalor syndrome [5]. Symptoms have been noted to include the following: extreme self-neglect of environment, health, and hygiene, excessive/abnormal hoarding (syllogomania), living in squalor, social withdrawal/living reclusively, refusal of help, and a distinct lack of concern and shame regarding one's living condition [6–10].

The current *Diagnostic and Statistical Manual of Mental Disorders 5th Edition* (*DSM-5*) does not list Diogenes syndrome as a psychiatric condition; however, it includes the closely related condition hoarding disorder ([11]; C.R. [3])—Diogenes is distinct from hoarding disorder, with the presence of squalor and neglect, without insight, distress, or emotional attachment differentiating it from syllogomania [2, 10, 12]. As noted by Lee and LoGiudice [12], the passive accumulation of rubbish and “failure to remove household waste is strictly speaking a form of neglect rather than hoarding” (p. 99). The importance of clear diagnostic criteria and classification is imperative to diagnose Diogenes accurately (for case examples highlighting the complexity and diversity of patients that may be incorrectly labelled as having Diogenes, see Lee and LoGiudice, 2012.). Indeed, the conditions leading to the hospitalisation and treatment of the patient reported in this case appeared circumstantially suggestive of hoarding disorder.

The literature reveals Diogenes syndrome to be highly comorbid with various psychiatric and somatic disorders, including depression, obsessive-compulsive disorder, personality disorder, and stress [1]. This case report offers a rare opportunity to better understand the distinction of Diogenes syndrome from hoarding disorder.

## 2. Case Report

A 51-year-old male was admitted to the hospital psychiatric ward following the police removing him from his home and taking him to the emergency room, where he was seen by the staff and assessed by the on-call psychiatrist. Written informed consent was obtained from the patient and formally witnessed, both prior to writeup and submission for publication.

Police officers attended the man's home following the alarm being raised by his stepfather regarding his welfare. It was reported that the man had not been seen or heard from in 3 weeks and that there was a large pile of unopened mail outside his door. Attempts were made to raise the man's attention by knocking loudly on the front door, but there was no response. On arrival to the property, the police officers noticed a strong-smelling odour coming from underneath the front door, and upon looking through the door windowpanes, could see flies on the other side. The front door being the only point of entry, the police forced it open.

**Figure 1:**
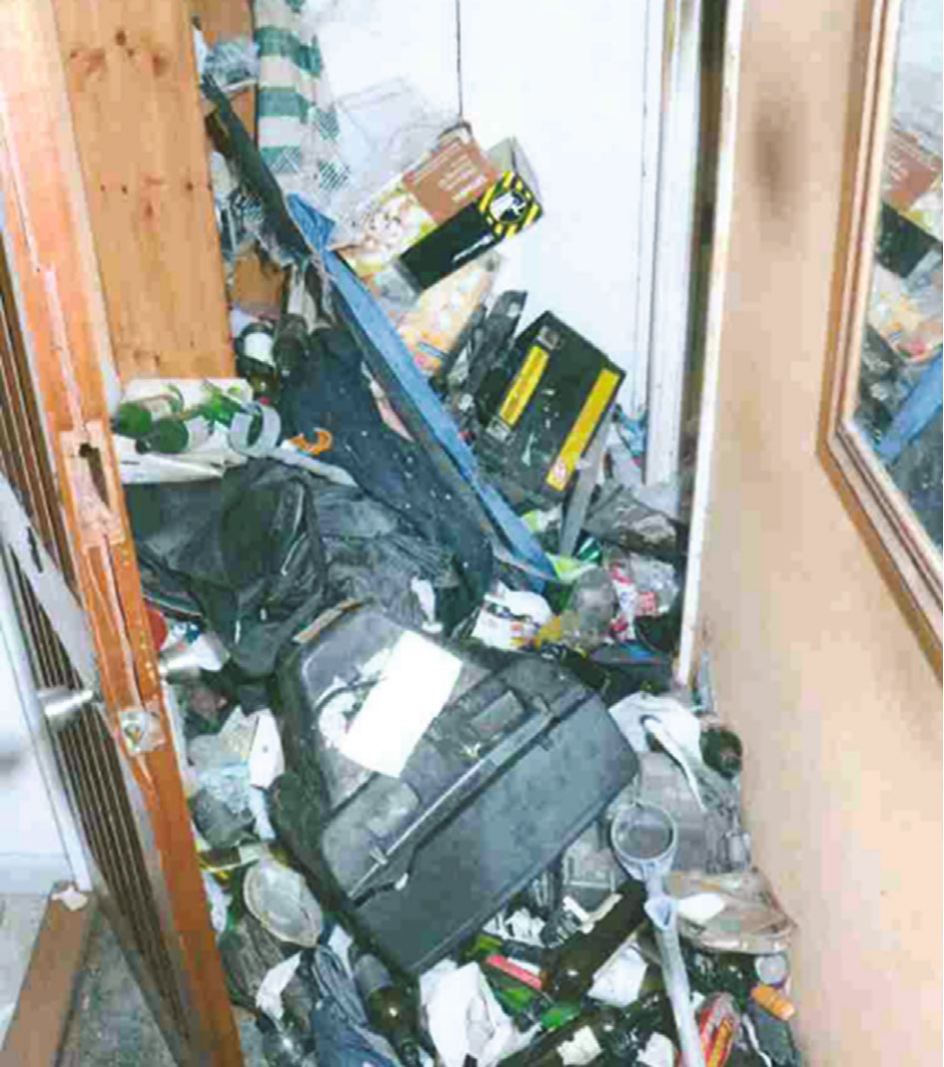
Entrance way.

**Figure 2:**
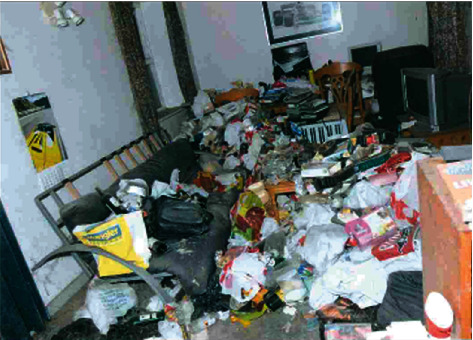
Living room.

Police officers squeezed through the door and climbed over a large amount of rubbish and decaying debris, including empty bottles, rotting take-out boxes, and cat food (Figure 1). The officers made their way down the hallway and, as they approached the living room, entered it, and found the man lying on top of a pile of rubbish wrapped in a rug (Figure 2). The room was heavily littered, and there was an intense smell of rotting mould, urine, and faeces. Officers shone a light on him and confirmed that he was breathing. The home was extremely hazardous, and therefore, the officers took great care to assist the man, who was naked under the rug, until medical professionals arrived.

**Figure 3:**
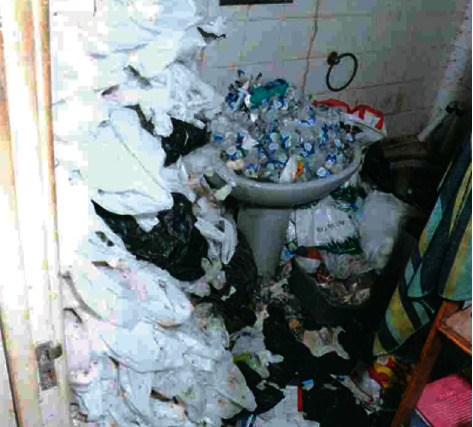
Bathroom.

**Figure 4:**
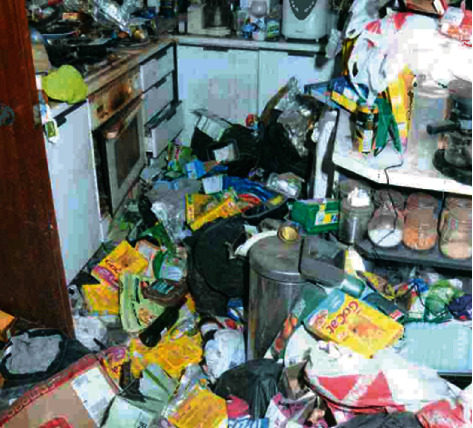
Kitchen.

The police searched the home for other individuals. Police officers found it difficult to move around the property due to the excessive amount of rubbish and debris—as they walked “bottles cracked under their feet”. Empty bottles and rubbish filled the sink, toilet, and bath (Figure 3). It appeared that the toilet no longer worked and that the individual had been using small plastic bags to defecate in, which he stored in the corner of the room—the police found hundreds of bags of faeces. In the living room, the ceiling was covered with a layer of thick cobwebs. “The windows were crusted over, and the garden outside though barely visible was clearly overgrown. The pots and pans in the kitchen were stacked high and unwashed with leftover food, mould, and maggots all over them” (Figure 4). The apartment was covered throughout in several feet of rubbish. In the living room, officers discovered plates of dried and decayed cat food that were hardly recognisable—the floor moved underneath the police officers' feet from rats running under the debris. The local authorities provided information contained in this section, used with permission.

Upon conferring with staff at emergency and the on-call psychiatrist, the man was deemed in an unfit mental state to care for himself and admitted to the hospital psychiatric ward. Bloods and physical examination were unremarkable, except that he had long and curled onychogryphotic toenails, which caused his feet to bend when walking, and cerumen impaction (excessive earwax) of the right ear causing partial deafness.

Initially, a medication-free assessment including a sleep chart, mood chart, and the Brief Psychiatric Rating Scale (BPRS) [13] was undertaken. Extended assessment while on the ward included the Beck Depression Inventory-II (BDI-II) [14] and Clinical Outcomes in Routine Evaluation (CORE-10) [15]. Scores revealed severe depression without secondary mental illness—BDI-II, severe depression, score 38 (range 29-63); BPRS, moderate anxiety and conceptual disorganisation, moderately severe depressive mood, and severe guilty feelings, scores of 4, 5, and 6 respectfully; and CORE-10, mild psychological global distress, clinical score of 13 (range 10-15).

On admission, mental state examination (MSE) revealed no evidence of any psychotic phenomenon—no evidence of hallucination, delusion, or thought disorder. Initially, the patient exhibited poor appetite and low sociability. He expressed suicidal ideation with a plan, but without intent—thoughts he described as having been present for many years, notable observed flat mood, vacant expression, and low ability to focus on tasks. He described his mood as “low” and appeared withdrawn with minimal interaction with staff. The patient initially stated a belief that people could read and/or insert thoughts into his mind; however, he clarified this by stating that people who knew him could take advantage of him and influence his life, resulting in him blocking everyone on his contact list on his mobile. Extended assessment revealed a depression score in the severe range; however, observed presentation was unrepresentative of the scale score, and routine reports indicated steadily increasing mood. Pharmacological treatment for psychosis was not indicated; however, discussions were held with regard to commencing an antidepressant, which the patient refused—patient deemed to have capacity with regard to pharmacological treatment for depression.

The patient reported isolating himself at home to avoid uncomfortable feelings experienced when with others and episodes of dissociation—e.g., drifting off and discovering that 1/2 hour has passed. The patient noted having difficulty reading, reporting rereading of book pages over and over again. A psychological therapy assessment was undertaken, and he was offered a referral to psychology and support from key workers within mental health. Upon discharge from the ward, he was followed up by secondary care mental health services for psychotherapy.

## 3. Discussion

### 3.1. Distinction from Hoarding Disorder

The circumstances of admission in this case appeared suggestive of hoarding disorder. However, although clear evidence of persistent difficulty discarding or parting with possessions (criterion A) was present, there was no reported perceived need to save the items or any distress associated with discarding them (criterion B). Further, although the associated difficulty in discarding the possessions congested living areas and compromised their intended use (criterion C), the patient had maintained occupational functioning (employed until 8 months prior) for 6 years with minimal personal social contact, without his living circumstance causing clinically significant distress until acute (criterion D).

This case was atypical of hoarding disorder. There was no reported early signs of nonbizarre collecting behaviour, no reported difficulty with the removal of possessions, no presence of bizarre behaviour while detained on the ward, and no attachment of meaning to the items collected. Further, the accumulation of bizarre items is noted as being more common in the context of obsessive-compulsive disorder but be very unusual in hoarding disorder. Typical features of hoarding disorder included indecisiveness, early traumatic life events, item collection precluding the use of space, and anxiety over what to do or throw away. Therefore, further investigation and assessment was undertaken to determine whether the hoarding was attributable to another medical condition (criterion E) and if the hoarding could be better explained by another medical condition (criterion F).

Diogenes syndrome, which is characterised by extreme self-neglect, domestic squalor, symptoms of catatonia, social withdrawal, apathy, compulsive hoarding of rubbish, and lack of concern with regard to situation, and executive dysfunction, which is characterised by difficulties in planning, decision making, and carrying out daily tasks, were considered as possible differential diagnoses.

Given that the mechanisms of Diogenes may not be readily obvious, a neuropsychological evaluation helps identify contributing factors [16]. Further, passive pathological accumulation is not exclusive to Diogenes, as it may also appear in patients with autism, dementia, or psychotic disorders [17, 18]. The autism spectrum quotient short form (AQ-10) [19] was used to screen for autistic spectrum disorder (ASD) traits—score of 7 (cut − off ≥ 6). Autism diagnostic testing results revealed ASD traits associated with social communication and interaction; however, scores were not suggestive of a diagnosis of ASD. Therefore, the patient was recommended for further neurological investigation (computerised tomography scan) to investigate any other organic causes for difficulties in executive functioning. Computerised tomography (CT) scan results indicated mild generalised cerebral atrophy consistent with patient's age. Similarly, scores from the Addenbrooke's Cognitive Examination-III (ACE-III) [20] and Frontal Lobe Assessment Battery (FAB) [21] were not indicative of cognitive impairment or early-onset dementia. Psychotic disorder was deemed not to be present by psychiatry, with MSE not indicative of psychosis. Patient's overall presentation improved steadily in conjunction with the personal care and psychological treatment received while hospitalised.

Scores from the Personality Inventory for DSM-5 (PID-5) [22] indicated personality dysfunction in the domains of negative affect (emotional lability, anxiousness, and separation insecurity) and detachment (withdrawal, anhedonia, and intimacy avoidance).

### 3.2. Treatment, Outcome, and Follow-Up

The patient was provided with psychotherapeutic intervention over the course of a two-year period, during which investigation and assessment into the underlying cause of his initial presentation was also undertaken. Given the complex history and presentation of the case, a combined integrative approach to psychotherapy was undertaken. An integrative psychotherapeutic approach was deemed appropriate in facilitating integration, wholeness, and personal growth for this patient—specifically, enabling him to move away from his objective perception of himself towards a subjective integrated experience, enabling him to make sense of the manifest symptoms represented by his physical environment and bodily state prior to hospitalisation.

Pharmacological treatment was initiated during psychotherapy following a review indicating a deterioration in patient's mood. Sertraline (50 mg daily) was commenced 8 months into treatment; however, the patient was not concordant and discontinued medication after 1 month—mood improved significantly without further medication. The patient had previously been prescribed Diazepam (15 mg daily) for an 8-day period 7 months into treatment for acute stress; however, he experienced and overly sedative effect, and therefore, this was discontinued.

At the time of discharge, the patient was receptive to ongoing assistance and was working cooperatively with mental health services with regard to his long-term self-care. Given the significant risk of him returning to a squalid living situation over time, an occupational therapy assessment was conducted prior to discharge providing him with recommendations with regard to his independent living going forward. Final assessments indicated borderline level of anxiety, normal level of depression (Hospital Anxiety and Depression Scale [23], score of 8 and 2 respectfully), a low level of psychological distress (CORE-10, clinical score 6 [range 5-10]), and a moderately high level of mental well-being (Warwick-Edinburgh Mental Well-Being Scale [24], score 48 [range 14-70]).

### 3.3. Case Considerations

Given that there is no agreed-upon constellation of symptoms representative of Diogenes, it is inherently difficult to diagnose [10]. Indeed, symptoms such as extreme self-neglect, domestic squalor, hoarding, social withdrawal, lack of shame, and refusal of help can also be found in various psychological and psychiatric conditions (e.g., depression, obsessive-compulsive disorder, schizophrenia, dementia, personality disorder, and hoarding disorder) [12]. Diogenes patients have a reported 46% 5-year death rate, primarily resulting from secondary physical and medical conditions stemming from poor hygiene, malnutrition, infection, and injury [2, 25]. As noted by Ferry [8], and as evidenced in the case presented here, “Diogenes syndrome is often identified by chance (e.g., a person collapses and trapped in rubbish)” (p. 29). Further, the disorder appears to follow a “distinct sociodemographic profile where it is found that persons are usually single, aged, having average or above-average intelligence, and also having good income” ([7], p. 2; [26]).

Extreme self-neglect has been reported in individuals with high intelligence and prior successful careers, who begin to live in squalid conditions following sudden life circumstances [10]. For example, cases of sudden-onset self-neglect, hoarding of rubbish, spoiled food, and excreta have been reported following a diagnosis of life-threatening conditions [2, 27]. Given such extant cases, it is reasonable to consider that Diogenes may also follow significant life trauma (cf. [26]). For example, in this patient's case, his wife committed suicide, following him leaving her 1 year prior. He was found 6 years later lying on top of a pile of rubbish in his home. He claimed that no one had entered his apartment during the entire time he resided there. Accordingly, it has been hypothesised that Diogenes is a stress response in individuals with certain premorbid personality traits, such as being socially withdrawn or aloof [7, 10]. Nevertheless, given the broad psychosocial and health implications of this syndrome, it is clear that specialised psychotherapeutic and social support is required in the treatment of these cases [27].

It has also been argued that Diogenes is a manifestation of significant underlying executive function deficits or preexisting psychological disorders that have previously been undiagnosed ([6]; cf. A.C. [28]). In the case presented here, neuropsychological assessment of executive function did not indicate cognitive impairment or early dementia, nor did CT scan images reveal any significant features of note other than those naturally associated with patient's age. Interestingly, reflecting on his home's living conditions when he was found, the patient described feeling unable to make small decisions that continued to escalate until he could not cope and just “shut down.” The syndrome's occurrence as a primary condition, without an explanatory psychiatric disorder, has received attention [29, 30]. For example, Sadlier et al. [29] highlight the cooccurrence of Diogenes with underlying key features of ASD—which in the case of the patient presented here, history and presentation were suggestive but not conclusive for a differential diagnosis. The patient's psychosocial and developmental history revealed longstanding social withdrawal (introversion) and isolation, above-average intelligence without matched career or lifestyle outcomes, passive accumulation of waste and bizarre items (ejaculate [early adulthood], faeces [older adulthood]), and complex familial and interpersonal problems. Indeed, as noted by Khan, “further investigation into this psychopathological process will be important in considering it as a distinct diagnostic entity to be considered for future editions of the *DSM*” (p. 11).

### 3.4. Conclusions

The case presented here provides us with a rare opportunity to consider a primary case of Diogenes syndrome in a nonelderly male patient. This case represents the known features of Diogenes syndrome and highlights the importance of distinguishing it from other related disorders, such as obsessive-compulsive disorder and obsessive-compulsive personality disorder [31]. Moreover, similar to other known cases, this case appears to support Karl Jaspers' formulation of the “social breakdown of the elderly” [30]. As noted by Badr et al. [31]:

[Karl Jaspers] proposed that this condition does not constitute a newly occurring psychopathological entity, as the whole picture is understandable from each subject's personality and stressful life events. He emphasised that the characteristics of the premorbid personality play an integral role in the pathogenesis of the syndrome. His view of this syndrome was that it represents a lifelong subclinical personality disorder, probably of a schizoid or paranoid type, that turns gradually into gross self-neglect and social isolation. This deterioration is precipitated by stressful life events, such as a loss of a spouse or ageing by itself, and is further aggravated by increasingly debilitating physical problems. Karl Jaspers called the social breakdown of the elderly “a personality-based abnormal emotional reaction development or adjustment disorder.” He explained that the complex cycle of personality factors, loneliness, stress, and somatic illness form a vicious cycle, resulting in a reclusive lifestyle, abandonment of basic social norms, and persistent refusal of help as they invoke the defense mechanisms of withdrawal and denial of need. (p. 12).

Additional considerations into the psychopathological process necessarily include examining the potential psychological and social “benefits” of the behaviours and their meaningfulness for the individual. For example, in the case presented here, the state of his home will have secured and maintained a cycle of psychosocial avoidance—protecting him from having to invite people in, thereby validating his reasons for isolation, and securing avoidance of the inherent disappointment of engaging in relationships, particularly intimate ones. Like many reported cases of Diogenes syndrome, the patients seek primarily to protect themselves from anxieties associated with the characteristics of their premorbid personality (cf. [32]). Indeed, a limitation in reporting this patient's case was the lack of full medical, familial, developmental, and psychosocial history.

The cause and development of Diogenes syndrome raise many questions. It is associated with various psychiatric and somatic disorders; however, investigations enabling the creation of clear diagnostic criteria and classification remains lacking. Importantly, this case highlights key features of Diogenes syndrome typical of all known primary presentations of this disorder, including passive accumulation of waste, without insight, distress, or emotional attachment, executive functioning deficit, longstanding social withdrawal or isolation, above-average intelligence, stressful life events, and relationship difficulties. The creation of an agreed-upon constellation of symptoms representative of Diogenes is essential to creating a formal *DSM* entry. It would facilitate the much-needed development of assessment measures to enable accurate diagnosis and treatment.

## Abbreviations

DSM: Diagnostic and Statistical Manual of Mental Disorders
BDI-II: Beck Depression Inventory-II
BPRS: Brief Psychiatric Rating Scale
CORE-10: Clinical Outcomes in Routine Evaluation
MSE: Mental state examination
AQ-10: Autism spectrum quotient short form
ASD: Autistic spectrum disorder
CT: Computerised tomography
ACE-III: Addenbrooke's Cognitive Examination
FAB: Frontal Lobe Assessment Battery
PID-5: A Personality Inventory for DSM-5.
